# Design, Synthesis, and Antitumor Activities of Some Novel Substituted 1,2,3-Benzotriazines

**DOI:** 10.3390/molecules13061427

**Published:** 2008-06-24

**Authors:** Jin-Ling Lv, Rui Wang, Dan Liu, Gang Guo, Yong-Kui Jing, Lin-Xiang Zhao

**Affiliations:** 1 Shenyang Pharmaceutical University, Shenyang, 110016, P.R. China; E-mails: jinlinglv12@yahoo.com.cn (Lv); ruiwang36@yahoo.com.cn (Wang); sammyld@163.com (Liu); guogang19761126@sina.com (Guo); 2 Mount Sinai School of Medicine, One Gustave L. Levy Place, New York, NY 10029, USA; E-mail: yongkui.jing@mssm.edu

**Keywords:** 1,2,3-Benzotriazine, synthesis, antiproliferative activity, vatalanib succinate

## Abstract

A series of novel substituted 1,2,3-benzotriazines based on the structures of vatalanib succinate (PTK787) and vandetanib (ZD6474) were designed and synthesized. The antiproliferative effects of these compounds were tested on microvascular endothelial cells (MVECs) using the MTT assay. Introduction of a methoxy and a 3-chloropropoxy group into the 1,2,3-benzotriazines increased the antiproliferative effects. 4-(3-Chloro-4-fluoroanilino)-7-(3-chloropropoxy)-6-methoxy-1,2,3-benzotriazine (**8m**) was the most effective compound. It was 4-10 fold more potent than PTK787 in inhibiting the growth of T47D breast cancer cells, DU145 and PC-3 prostate cancer cells, LL/2 murine Lewis lung cancer cells and B16F0 melanoma cells.

## Introduction

**Figure 1:**
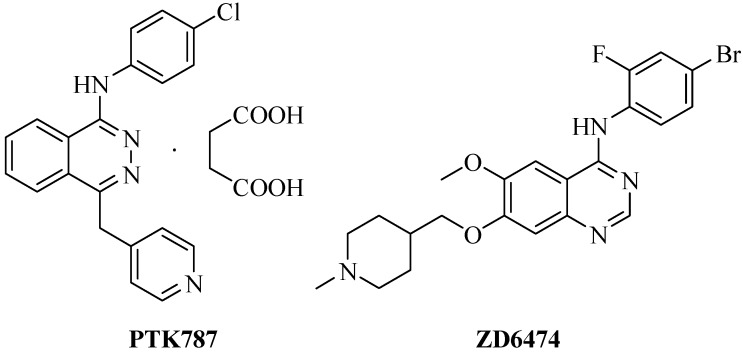
Structures of vatalanib succinate (PTK787) and vandetanib (ZD6474).

Vascular endothelial growth factor (VEGF) plays an important role in both physiological and pathological angiogenesis by binding to its receptor, VEGFR [1]. The expression of VEGFR is increased in the majority of cancers and is associated with survival, migration and invasion of solid tumor cells [1]. Elevated VEGF levels have been found to increase resistance to chemotherapy [2, 3] and VEGF signaling has been used as a therapeutic target for the treatment of cancer. VEGFR inhibitors such as vatalanib succinate (PTK787/ZK222584) and vandetanib (ZD6474) have been used in clinical trials in several types of cancer [4,5,6,7]. Although both agents showed antitumor activities alone, combinations with chemotherapeutic agents seem to be required for reaching therapeutic effects [8,9,10,11,12]. To improve the antiproliferative abilities of PTK787 and ZD6474 (Figure 1), we have analyzed the structures of PTK787 and ZD6474. PTK787 contains 2,3-benzodizine and ZD6474 contains 1,3-benzodizine. We thought that the replacement of the benzodizine with 1,2,3-benzotriazine might have increased activities compared to PTK787. Thus, we designed and synthesized eighteen novel compounds (**8a-m**) with a basic 1,2,3-benzotriazine scaffold and the introduction of a methoxy group or a short alkoxy group. The antiproliferative effects of these compounds were determined in microvascular endothelial cells (MVECs) using MTT assay and the antiproliferative effects of compound **8m** were found to be the most profound in inhibiting growth of MVECs. Therefore, it was further investigated in T47D breast cancer cells, DU145 and PC-3 prostate cancer cells, LL/2 murine Lewis lung cancer cells and B16F0 melanoma cells.

## Results and Discussion

### Design and synthesis of novel 1,2,3-benzotriazines

Recently, it was reported that 1,2,4-benzotriazines were inhibitors of Src and the binding model of 1,2,4-benzotriazines with Src showed the importance of the N1 and N2 atoms [13, 14]. We considered that VEGFR and Src might react with substrates in a similar fashion as both of them are tyrosine kinases. Both PTK787 and ZD6474 contain a substituted anilino group. ZD6474 contains a methoxy group at the C6 position and a short side chain at the C7 position. We thought that these substituents are required for the antitumor activity. Thus, we decided using 1,2,3-benzotriazine to replace the phthalazine in PTK787 or the quinazoline in ZD6474 and to add a methoxy group at the C6 position and a short alkoxy group at the C7 position of 1,2,3-benzotriazine. A series of target compounds, **8a-r,** were obtained and their structures are listed in Table 1.

**Scheme 1:**
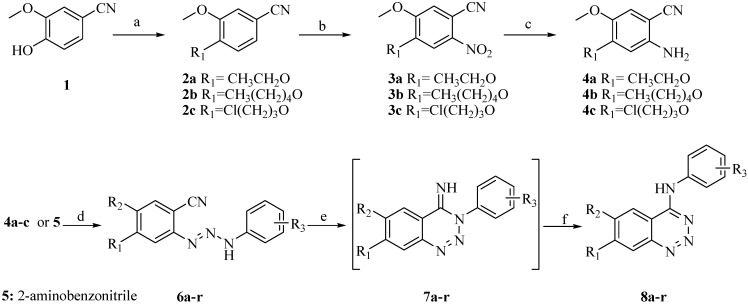
Synthetic route to the target compounds.

Compounds **8a-r** were prepared according to the synthetic route outlined in Scheme 1. 4-Cyano-2-methoxyphenol (**1**) reacted with various alkyl halides to afford intermediates **2a-c **in satisfactory yields. Compounds **2a-c** were selectively nitrated at 30 ^o^C to give nitro compounds **3a-c** in 87-95% yields. The reduction of the nitro compounds **3a-c** to the corresponding amines **4a-c** was catalyzed by Pd/C [15]. Compounds **4a-c** were diazotized and then coupled with substituted anilines at 0 ^o^C, followed by chromatographic purification to afford triazenes **6b-r**. Compound **6a** was prepared by the coupling of diazotized 2-aminobenzonitrile (**5**) and 4-chloroaniline. The cyclization of compounds **6a-r** in 70% ethanol formed intermediates **7a-r**, which then rearranged to compounds **8a-r** after refluxing in acetic acid [16]. As intermediates **7a-r** were unstable, they were used directly in the next step without further purification.

*Reagents and conditions:* (a) alkyl halides, DMF, K_2_CO_3_, 37^ o^C; (b) HNO_3_, 30^ o^C; (c) Pd/C, cyclohexene, ethanol, reflux; (d) NaNO_2_/HCl, 0^ o^C; substituted aniline, 0^ o^C; (e) 70% ethanol, reflux; (f) AcOH, reflux.

### Antiproliferative activities in MVECs

The antiproliferative effects of compounds **8a-r** in MVECs were determined (Table 1) and the structure-activity relationships were analyzed. The results revealed that compounds with a methoxy group (R_2_) at the C6 position and an alkoxy group (R_1_) at the C7 position (**8b-r**) had an increased activity compared to that of compound **8a** which did not have substitutions at the C6 and C7 positions. The compounds with a 3-chloropropoxy group (R_1_) at the C7 position (**8h-r**) were more active than compounds with an ethoxy group (**8b-d**) or a pentyloxy group (**8e-g**).

By comparing the activities of compounds with a methoxy group at the C6 position and a 3-chloropropoxy group at the C7 position, but with a different substituted group (R_3_) at the C4 anilino group (**8h-r**), it revealed that a substitution at the C4 anilino increased the antiproliferative activity in MVECs compared to compound **8r**, which did not have a substitution at the C4 anilino group. The electronic effect of a substituent on the anilino group did not influence the antiproliferative activities (comparing compounds **8k-o**). Compounds with two substituents at the C3’ and C4’ positions of the C4 anilino group (**8i**, **8m,** and **8p**) were more active than compounds with two substituents at the C3’ and C5’ positions (**8h**) or compounds with one substituent (**8j**, **8k**, **8l**, **8n**, **8o**, **8q**). Compound **8m** was the most potent one in inhibiting proliferation of MVECs with a GI_50_ value of 7.98 μM. The compounds with substituents at the anilino group (compounds **8h-q**) were more effective than PTK787 in inhibiting growth of MVECs after the introduction of a methoxy group at the C6 position and a 3-chloropropoxy group at the C7 position.

**Table 1:** the structures of target compounds and tdeir antiproliferative effects in MVECs.

Compounds	R_1_	R_2_	R_3_	GI_50_ (μM)^ a^
**8a**	H	H	4-Cl	>80
**8b**	CH_3_CH_2_O	CH_3_O	4-Cl	26.54±1.43
**8c**	CH_3_CH_2_O	CH_3_O	4-CH_3_	42.56±2.79
**8d**	CH_3_CH_2_O	CH_3_O	3-Cl, 4-F	38.81±1.86
**8e**	CH_3_(CH_2_)_4_O	CH_3_O	4-Cl	25.35±1.02
**8f**	CH_3_(CH_2_)_4_O	CH_3_O	3-OCF_3_	42.64±2.27
**8g**	CH_3_(CH_2_)_4_O	CH_3_O	4-OCF_3_	37.98±1.98
**8h**	Cl(CH_2_)_3_O	CH_3_O	3,5-di-Cl	28.84±1.40
**8i**	Cl(CH_2_)_3_O	CH_3_O	3,4-di-Cl	11.02±0.49
**8j**	Cl(CH_2_)_3_O	CH_3_O	4-Cl	18.59±1.31
**8k**	Cl(CH_2_)_3_O	CH_3_O	4-CH_3_	17.08±0.65
**8l**	Cl(CH_2_)_3_O	CH_3_O	4-F	23.05±0.92
**8m**	Cl(CH_2_)_3_O	CH_3_O	3-Cl, 4-F	7.98±0.35
**8n**	Cl(CH_2_)_3_O	CH_3_O	3-OCF_3_	24.86±0.86
**8o**	Cl(CH_2_)_3_O	CH_3_O	4-OCF_3_	23.19±1.12
**8p**	Cl(CH_2_)_3_O	CH_3_O	3-CF_3_, 4-F	15.22±0.51
**8q**	Cl(CH_2_)_3_O	CH_3_O	3-CF_3_	21.38±1.54
**8r**	Cl(CH_2_)_3_O	CH_3_O	H	>80
vatalanib succinate				38.15±2.07
(PTK787)				

^a^^. ^GI_50_ is the concentration that inhibits 50% of cell growth. The cells were treated with various concentrations of the tested compounds for 4 days and cell growth inhibition was determined using the 3-(4,5-dimethylthiazol-2-yl)-2,5-diphenyltetrazolium bromide (MTT) assay. Data shown are means ± SD of three independent experiments.

The antiproliferative effects of compound **8m** were then tested in tumor cell lines. The antiproliferative effects of compound **8m** were determined in human T47 breast cancer cells, DU-145 and PC-3 prostate cancer cells, murine LL/2 Lewis lung cancer cells and B16F0 melanoma cells using MTT assay (Table 2). Compound **8m** was more effective than PTK787 to inhibit cell growth in all the tested cell lines.

**Table 2:** Antiproliferative effects of compound **8m** and PTK787 in several tumor cell lines.

Compounds	GI_50_ (μM) ^a^				
	T47D	DU-145	PC-3	LL/2	B16F0
**8m**	5.04±0.32	4.96±0.43	6.91±0.30	3.79±0.19	5.19±0.22
**PTK787**	36.32±1.88	41.28±2.47	63.68±3.55	31.15±2.01	18.71±1.67

^a^^. ^Cells were treated with various concentrations of the tested compounds for 4 days and the cell growth inhibition was determined using the MTT assay. Data shown are means ± SD of three independent experiments.

## Conclusions

In summary, our data indicate that 1,2,3-benzotriazines substituted with a methoxy group at the C6 position and a 3-chloropropoxy group at the C7 position exhibit antiproliferative activities. Most of these compounds are more effective than PTK787 in inhibiting proliferation of MVECs. Compound **8m** is the most potent and exhibits greater anti-tumor cell growth activity activities than PTK787. Since we did not stimulate the cell growth of MVECs and tumor cells with VEGF, the observed antiproliferative effects of these compounds, including PTK787, may be through a pathway independent of VEGFR inhibition. Because of the high similarity of 1,2,3-benzotriazine and 1,2,4-benzotriazine, the increased antiproliferative effects of the designed compounds in tumor cells may be due to the inhibition of Src, or other tyrosine kinases which is not inhibited by PTK787 [17]. The effects of our novel 1,2,3-benzotriazines on the activities of VEGFRs, Src and other kinases are under investigation.

## Experimental

### General

Reagents (analytical grade) were obtained from commercial suppliers and used without further purification unless otherwise noted. ^1^H- and ^13^C-NMR spectra were recorded on a Bruker ARX-300 instrument with tetramethylsilane as the internal standard. IR spectra were recorded on a Bruker IR-27G spectrometer. MS were determined on either Finnigan MAT/USA spectrometer (LC-MS). Elemental analysis was determined on a Carlo-Erba 1106 Elemental analysis instrument (Carlo Erba, Milan, Italy). The melting points were determined on an electrically heated X4 digital visual melting point apparatus and were uncorrected. The structures of the synthesized compounds were characterized by melting point (mp), infrared (IR) spectra, nuclear magnetic resonance (NMR) spectra and mass spectral (MS) data.

### General procedure for the synthesis of 4-substituted-3-methoxybenzonitriles **2a-c**

A solution of 4-cyano-2-methoxyphenol (**1**, 1.00 g, 6.70 mmol) in anhydrous DMF (4.00 mL) was stirred and cooled with a water bath. K_2_CO_3_ (1.39 g, 10.1 mmol) was added and the mixture was stirred at 20 ^o^C for 1 h. The corresponding alkyl halide (8.13 mmol) was added dropwise, the mixture was stirred at room temperature overnight, then heated at 37 ^o^C for 6 h and finally poured into a mixture of ice/H_2_O (100 mL). After stirring for 10 min, a precipitate was formed. It was filtered off, washed with H_2_O, and air-dried to yield the 4-substituted-3-methoxybenzonitriles **2a-c** as white solids.

*4-Ethoxy-3-methoxybenzonitrile* (**2a**): Yield: 91.8%; mp: 102-103 ^o^C. ^1^H-NMR (DMSO-*d_6_*) δ: 7.36 (2H, m, H-2, H-6), 7.04 (1H, s, H-5), 4.11 (2H, q, *J* = 6.9 Hz, CH_3_CH_2_O-), 3.81 (3H, s, -OCH_3_), 1.35 (3H, t, *J* = 6.9 Hz, CH_3_CH_2_O-); LC-MS: 178.1 (M+H)^+^; Anal. Calcd. for C_10_H_11_NO_2_: C 67.78, H 6.26, N 7.90; Found: C 67.79, H 6.24, N 7.91.

*3-Methoxy-4-pentyloxybenzonitrile* (**2b**): Yield: 89.0%; mp: 55-56 ^o^C. ^1^H-NMR (DMSO-*d_6_*) δ: 7.37 (2H, m, H-2, H-6), 7.09 (1H, d, *J* = 8.1 Hz, H-5), 4.02 (2H, t, CH_3_(CH_2_)_3_CH_2_O-), 3.79 (3H, s, -OCH_3_), 1.71 (2H, m, CH_3_CH_2_CH_2_CH_2_CH_2_O-), 1.34 (4H, m, CH_3_CH_2_CH_2_CH_2_CH_2_O-), 0.88 (3H, t, *J* = 6.9 Hz, CH_3_CH_2_CH_2_CH_2_CH_2_O-); LC-MS: 220.1 (M+H)^+^; Anal. Calcd. for C_1__3_H_1__7_NO_2_: C 71.21, H 7.81, N 6.39; Found: C 71.20, H 7.80, N 6.39.

*4-(3-Chloropropoxy)-3-methoxybenzonitrile* (**2c**): Yield: 87.2%. ^1^H-NMR (DMSO-*d_6_*) δ: 7.41 (2H, m, H-2, H-6), 7.15 (1H, d, *J* = 8.8 Hz, H-5), 4.16 (2H, t, *J* = 6.0 Hz, ClCH_2_CH_2_CH_2_O-), 3.79 (5H, m, -OCH_3_, ClCH_2_CH_2_CH_2_O-), 2.20 (2H, m, ClCH_2_CH_2_CH_2_O-); LC-MS: 226.1 (M+H)^+^; Anal. Calcd. for C_1__1_H_1__2_ClNO_2_: C 58.54, H 5.36, N 6.21; Found: C 58.52, H 5.37, N 6.20.

### General procedure for the synthesis of 4-substituted-5-methoxy-2-nitrobenzonitriles, **3a-c**

A solution of 4-substituted-3-methoxybenzonitriles **2** (9.10 mmol) in nitric acid (10 mL) was heated to 30 ^o^C for 2 h, poured into ice-water (100 mL), filtered, and washed with water to afford compounds **3 **as light yellow solids.

*4-Ethoxy-5-methoxy-2-nitrobenzonitrile* (**3a**): Yield: 88.1%; mp: 198-199 ^o^C. ^1^H-NMR (DMSO-*d_6_*) δ: 7.85 (1H, s, H-3), 7.70 (1H, s, H-6), 4.25 (2H, q, *J* = 6.9 Hz, CH_3_CH_2_O-), 3.97 (3H, s, -OCH_3_), 1.37 (3H, t, *J* = 6.9 Hz, CH_3_CH_2_O-); LC-MS: 223.1 (M+H)^+^; Anal. Calcd. for C_1__0_H_10_N_2_O_4_: C 54.05, H 4.54, N 12.61; Found: C 54.03, H 4.55, N 12.60.

*5-Methoxy-2-nitro-4-pentyloxybenzonitrile* (**3b**): Yield: 92.9%; mp: 136-137 ^o^C. ^1^H-NMR (DMSO-*d_6_*) δ: 7.89 (1H, s, H-3), 7.80 (1H, s, H-6), 4.16 (2H, t, *J* = 6.3 Hz, CH_3_CH_2_CH_2_CH_2_CH_2_O-), 3.95 (3H, s, -OCH_3_), 1.72 (2H, m, CH_3_CH_2_CH_2_CH_2_CH_2_O-), 1.35 (4H, m, CH_3_CH_2_CH_2_CH_2_CH_2_O-), 0.89 (3H, t, *J* = 6.9 Hz, CH_3_CH_2_CH_2_CH_2_CH_2_O-); LC-MS: 265.1 (M+H)^+^; Anal. Calcd. for C_1__3_H_1__6_N_2_O_4_: C 59.08, H 6.10, N 10.60; Found: C 59.10, H 6.11, N 10.59.

*4-(3-Chloropropoxy)-5-methoxy-2-nitrobenzonitrile* (**3c**): Yield: 95.3%; mp: 133-134 ^o^C. ^1^H-NMR (DMSO-*d_6_*) δ: 7.91 (1H, s, H-3), 7.71 (1H, s, H-6), 4.32 (2H, t, *J* = 6.0 Hz, ClCH_2_CH_2_CH_2_O-), 4.00 (3H, s, -OCH_3_), 3.78 (2H, t, *J* = 6.3 Hz, ClCH_2_CH_2_CH_2_O-), 2.23 (2H, m, ClCH_2_CH_2_CH_2_O-); LC-MS: 271.0 (M+H)^+^; Anal. Calcd. for C_1__1_H_1__1_ClN_2_O_4_: C 48.81, H 4.10, N 10.35; Found: C 48.82, H 4.11, N 10.34.

### General procedure for the synthesis of 4-substituted-2-amino-5-methoxybenzonitriles, **4a-c**

A mixture of compound **3** (3.69 mmol) and Pd/C (10%, 7.00 g) in anhydrous ethanol (10.0 mL) was stirred and heated under reflux. Cyclohexene (2.30 mL, 22.6 mmol) was added dropwise. The mixture was refluxed overnight, then cooled to 40 °C, filtered, and washed with ethanol. The filtrate was concentrated to yield a solid. The crude product was suspended in ethanol, stirred at 40 °C for 30 min, cooled to room temperature, filtered to produce pure compound **4** as a yellow solid.

*2-Amino-4-ethoxy-5-methoxybenzonitrile* (**4a**): Yield: 49.1%; mp: 135-136 °C. 1H-NMR (DMSO-*d_6_*) δ: 6.87 (1H, s, H-6), 6.39 (1H, s, H-3), 5.58 (2H, s, NH2), 3.96 (2H, q, *J* = 6.9 Hz, CH_3_CH_2_O-), 3.65 (3H, s, -OCH_3_), 1.33 (3H, t, *J* = 6.9 Hz, CH_3_CH_2_O-); LC-MS: 193.1 (M+H)+; Anal. Calcd. for C_10_H_12_N_2_O_2_: C 62.49, H 6.29, N 14.57; Found: C 62.50, H 6.30, N 14.58.

*2-Amino-5-methoxy-4-pentyloxybenzonitrile* (**4b**): Yield: 50.1%; mp: 136-137 °C. 1H-NMR (DMSO-*d_6_*) δ: 6.85 (1H, s, H-6), 6.38 (1H, s, H-3), 5.57 (2H, s, NH2), 3.88 (2H, t, J = 6.3 Hz, CH_3_CH_2_CH_2_CH_2_CH_2_O-), 3.63 (3H, s, -OCH3), 1.70 (2H, m, CH_3_CH_2_CH_2_CH_2_CH_2_O-), 1.34 (4H, m, CH_3_CH_2_CH_2_CH_2_CH_2_O-), 0.88 (3H, t, J = 6.9 Hz, CH_3_CH_2_CH_2_CH_2_CH_2_O-); LC-MS: 235.1 (M+H)+; Anal. Calcd. for C13H18N2O2: C 66.64, H 7.74, N 11.96; Found: C 66.62, H 7.73, N 11.97.

*2-Amino-4-(3-chloropropoxy)-5-methoxybenzonitrile* (**4c**): Yield: 47.2%; mp: 118-119 oC. 1H-NMR (DMSO-*d_6_* δ: 6.89 (1H, s, H-6), 6.43 (1H, s, H-3), 5.62 (2H, s, NH2), 4.03 (2H, t, *J* = 6.1 Hz, ClCH_2_CH_2_CH_2_O-), 3.77 (2H, t, *J* = 6.4 Hz, ClCH_2_CH_2_CH_2_O-), 3.65 (3H, s, -OCH3), 2.18 (2H, m, ClCH_2_CH_2_CH_2_O-); LC-MS: 241.1 (M+H)+; Anal. Calcd. for C_11_H_13_ClN_2_O_2_: C 54.89, H 5.44, N 11.64; Found: C 54.88, H 5.45, N 11.63.

### General procedure for the synthesis of 7-alkoxy-6-methoxy-4-substituted-1,2,3-benzotriazines **8a-r**

Compound **4** (or compound **5** when preparing **8a**, 10.0 mmol) in 10N-hydrochloric acid (30.0 mL) was cooled to 0 ^o^C and diazotized with sodium nitrite (0.71 g) in water (10.0 mL). The diazonium solution was neutralized with excess of sodium acetate trihydrate and stirred for 2 h at 0 ^o^C with a corresponding substituted aniline (10.0 mmol). The solution was kept overnight at 4 ^o^C, filtered, and washed with water. The crude material was purified on a silica gel column with petroleum ether : ethyl acetate (v/v) = 10:1 to yield a yellow solid **6**. Compound **6 **was boiled in 70% ethanol (25.0 mL) for 1 h, then the solution was evaporated under reduced pressure to dryness. Acetic acid (10.0 mL) was added and the solution was refluxed for 2 h, cooled, poured into water (100 mL), filtered, and dried to afford 7-alkoxyl-6-methoxy-4-substituted-1,2,3-benzotriazines **8**.

*4-(4-chloroanilino)-1,2,3-benzotriazine* (**8a**)*:* Yield: 68.9%; mp: 230 ^o^C. IR (KBr): 3266, 3086, 1621, 1559, 1493, 1424, 1147, 766 cm^-1^; ^1^H-NMR (DMSO-*d_6_*) δ: 10.02 (1H, s, NH), 8.62 (1H, d, J = 8.1 Hz), 8.23 (1H, d, J = 8.1 Hz), 8.13 (1H, t, J = 8.2 Hz), 8.05 (1H, m), 7.90 (2H, m), 7.52 (2H, m); LC-MS: 257.1 (M+H)^+^; ^13^C-NMR (DMSO-*d_6_*) δ : 151.02, 143.53, 137.58, 134.73, 132.38, 128.66, 128.06, 127.47, 124.28, 121.87, 109.04; Anal. Calcd. for C_1__3_H_9_ClN_4_: C 60.83, H 3.53, N 21.83; Found: C 60.79, H 3.51, N 21.85.

*4-(4-Chloroanilino)-7-ethoxy-6-methoxy-1,2,3-benzotriazine* (**8b**): Yield: 72.1%; mp: 230 ^o^C. IR (KBr): 3296, 2928, 1612, 1560, 1494, 1423, 1291, 818 cm^-1^; ^1^H-NMR (DMSO-*d_6_*) δ: 9.68 (1H, s, NH), 7.92 (3H, m, H-8, H-2’, H-6’), 7.51 (3H, m, H-8, H-3’, H-5’), 4.29 (2H, q, *J* = 6.6 Hz, CH_3_CH_2_O-), 4.03 (3H, s, -OCH_3_), 1.44 (3H, t, *J* = 6.9 Hz, CH_3_CH_2_O-); LC-MS: 331.1 (M+H)^+^; ^13^C-NMR (DMSO-*d_6_*) δ: 154.22 (C-7), 153.27 (C-6), 150.12 (C-10), 141.65 (C-9), 137.95 (C-1’), 128.57, 127.48, 123.95, 106.78 (C-5), 103.84 (C-4), 100.09 (C-8), 64.74 (CH_3_CH_2_O-), 56.73 (-OCH_3_), 14.42 (CH_3_CH_2_O-); Anal. Calcd. for C_16_H_15_ClN_4_O_2_: C 58.10, H 4.57, N 16.94; Found: C 58.01, H 4.56, N 16.96.

*7-Ethoxy-6-methoxy-4-(4-methylanilino)-1,2,3-benzotriazine* (**8c**): Yield: 73.2%; mp: 249-251 ^o^C. IR (KBr): 33381, 2980, 1614, 1512, 1426, 1283, 1244, 1103, 856, 816 cm^-1^; ^1^H-NMR (DMSO-*d_6_*) δ: 9.50 (s, 1H, NH), 7.90 (s, 1H, H-8), 7.70 (d, 2H, *J* = 8.2 Hz, ArH-2’H, 6’H), 7.53 (s, 1H, H-5), 7.25 (d, 2H, *J* = 8.2 Hz, ArH-3’H, 5’H), 4.29 (q, 2H, *J* = 6.9 Hz, CH_3_CH_2_O-), 4.02 (s, 3H, CH_3_O-), 2.34 (s, 3H, ArH-4’CH_3_), 1.44 (t, 3H, *J* = 6.8 Hz, CH_3_CH_2_O-); LC-MS: 311.2 (M+H)^+^; ^13^C-NMR (DMSO-*d_6_*) δ: 154.16, 153.19, 150.30, 141.62, 138.86, 128.72, 124.04, 122.70, 106.77, 103.82, 100.12, 64.73, 56.72, 20.89, 14.46; Anal. Calcd. for C_17_H_18_N_4_O_2_: C 65.79, H 5.85, N 18.05; Found: C 65.68, H 5.81, N 18.07.

*4-(3-Chloro-4-fluoroanilino)-7-ethoxy-6-methoxy-1,2,3-benzotriazine* (**8d**): Yield: 64.1%; mp: 248-250 ^o^C. IR (KBr): 3288, 1609, 1554, 1500, 1417, 1292, 1237, 1222, 1106 cm^-1^; ^1^H-NMR (DMSO-*d_6_*) δ: 9.66 (s, 1H, NH), 8.17 (m, 1H, ArH-2’H), 7.87 (s, 1H, H-8), 7.82 (m, 1H, ArH-5’H), 7.58 (s, 1H, H-5), 7.51 (d, 1H, *J* = 9 Hz, ArH-6’H), 4.31 (q, 2H, *J* = 6.9 Hz, CH_3_CH_2_O-), 4.03 (s, 3H, CH_3_O-), 1.44 (t, 3H, *J* = 6.9 Hz, CH_3_CH_2_O-); LC-MS: 349.2 (M+H)^+^; ^13^C-NMR (DMSO-*d_6_*) δ: 154.43, 154.29, 153.62, 152.10, 150.35, 141.80, 136.65, 124.10, 123.00, 122.90, 119.55, 117.10, 116.80, 107.15, 104.13, 100.28, 64.69, 56.65, 14.46; Anal. Calcd. for C_16_H_14_ClFN_4_O_2_: C 55.10, H 4.05, N 16.06; Found: C 55.00, H 4.07, N 16.04.

*4-(4-Chloroanilino)-6-methoxy-7-pentyloxy-1,2,3-benzotriazine* (**8e**): Yield: 69.3%; mp: 213 ^o^C. IR (KBr): 3378, 2954, 1612, 1562, 1507, 1426, 1245, 817 cm^-1^; ^1^H-NMR (DMSO-*d_6_*) δ: 9.63 (1H, s, NH), 7.91 (3H, m, H-8, H-2’, H-6’), 7.53 (3H, m, H-8, H-3’, H-5’), 4.24 (2H, t, *J* = 6.0 Hz, CH_3_CH_2_CH_2_CH_2_CH_2_O-), 4.03 (3H, s, -OCH_3_), 1.82 (2H, m, CH_3_CH_2_CH_2_CH_2_CH_2_O-), 1.40 (4H, m, CH_3_CH_2_CH_2_CH_2_CH_2_O-), 0.92 (3H, t, *J* = 6.0 Hz, CH_3_CH_2_CH_2_CH_2_CH_2_O); LC-MS: 373.2 (M+H)^+^; ^13^C-NMR (DMSO-*d_6_*) δ: 154.31 (C-7), 153.27 (C-6), 150.30 (C-10), 141.63 (C-9), 138.88 (C-1’), 128.73, 127.40, 123.91, 106.72 (C-5), 103.82 (C-4), 100.05 (C-8), 68.98 (CH_3_CH_2_CH_2_CH_2_CH_2_O-), 56.79 (-OCH_3_), 28.17 (CH_3_CH_2_CH_2_CH_2_CH_2_O-), 27.78 (CH_3_CH_2_CH_2_CH_2_CH_2_O-), 21.98 (CH_3_CH_2_CH_2_CH_2_CH_2_O-), 14.04 (CH_3_CH_2_CH_2_CH_2_CH_2_O-); Anal. Calcd. for C_19_H_21_ClN_4_O_2_: C 61.21, H 5.68, N 15.03; Found: C 61.11, H 5.64, N 14.99.

*6-Methoxy-7-pentyloxy-4-(3-trifluoromethoxyanilino)-1,2,3-benzotriazine* (**8f**): Yield: 73.1%; mp: 181-183 ^o^C. IR (KBr): 3421, 2931, 1613, 1508, 1446, 1258, 1216, 1163, 1105, 845, 784 cm^-1^; ^1^H-NMR (DMSO-*d_6_*) δ: 9.72 (s, 1H, NH), 8.04 (s, 1H, ArH-2’H), 7.92 (d, 1H, *J* = 8.9 Hz, ArH-4’H), 7.91 (s, 1H, H-8), 7.60 (s, 1H, H-5), 7.56 (t, 2H, *J* = 8.6 Hz, ArH-5’H), 7.14 (d, 1H, *J* = 8.7 Hz, ArH-6’H), 4.24 (t, 2H, *J* = 6.3 Hz, CH_3_CH_2_CH_2_CH_2_CH_2_O-), 4.05 (s, 3H, CH_3_O-), 1.83 (m, 2H, CH_3_CH_2_CH_2_CH_2_CH_2_O-), 1.42 (m, 4H, CH_3_CH_2_CH_2_CH_2_CH_2_O-), 0.92 (t, 3H, *J* = 6.9 Hz, CH_3_CH_2_CH_2_CH_2_CH_2_O-); LC-MS: 423.2 (M+H)^+^; ^13^C-NMR (DMSO-*d_6_*) δ: 154.23, 153.52, 150.28, 148.76, 141.90, 141.06, 130.41, 124.25, 121.80, 118.10, 115.20, 114.04, 106.92, 104.11, 100.19, 68.98, 56.79, 28.17, 27.78, 21.98, 14.04; Anal. Calcd. for C_20_H_21_F_3_N_4_O_3_: C 56.87, H 5.01, N 13.26; Found: C 56.81, H 5.00, N 13.23.

*6-Methoxy-7-pentyloxy-4-(4-trifluoromethoxyanilino)-1,2,3-benzotriazine* (**8g**): Yield: 70.2%; mp: 230-232 ^o^C. IR (KBr): 3426, 2959, 1615, 1510, 1428, 1247, 1200, 1165, 1102, 850, 786 cm^-1^; ^1^H-NMR (DMSO-*d_6_*) δ: 9.69 (s, 1H, NH), 7.98 (d, 2H, *J* = 9.0 Hz, ArH-3’H, 5’H), 7.90 (s, 1H, H-8), 7.58 (s, 1H, H-5), 7.46 (d, 2H, *J* = 8.7 Hz, ArH-2’H, 6’H), 4.24 (t, 2H, *J* = 6.3 Hz, CH_3_CH_2_CH_2_CH_2_CH_2_O-), 4.04 (s, 3H, CH_3_O-), 1.83 (m, 2H, CH_3_CH_2_CH_2_CH_2_CH_2_O-), 1.42 (m, 4H, CH_3_CH_2_CH_2_CH_2_CH_2_O-), 0.92 (t, 3H, *J* = 6.9 Hz, CH_3_CH_2_CH_2_CH_2_CH_2_O-); LC-MS: 423.2 (M+H)^+^; ^13^C-NMR (DMSO-*d_6_*) δ: 154.31, 153.27, 150.30, 144.05, 141.54, 137.91, 125.24, 123.93, 122.14, 121.45, 106.86, 103.83, 100.08, 68.97, 56.76, 28.17, 27.76, 21.97, 14.04; Anal. Calcd. for C_20_H_21_F_3_N_4_O_3_: C 56.87, H 5.01, N 13.26; Found: C 56.88, H 5.03, N 13.27.

*7-(3-Chloropropoxy)-4-(3,5-dichloroanilino)-6-methoxy-1,2,3-benzotriazine* (**8h**): Yield: 72.9%; mp: 110-112 ^o^C. IR (KBr): 3430, 2919, 1620, 1508, 1423, 1289, 849 cm^-1^; ^1^H-NMR (DMSO-*d_6_*) δ: 9.74 (1H, s, NH), 8.10 (2H, d, *J* = 1.77 Hz, H-2’, H-6’), 7.91 (1H, s, H-8), 7.68 (1H, s, H-5), 7.37 (1H, s, H-4’), 4.39 (2H, t, *J* = 6.15 Hz, ClCH_2_CH_2_CH_2_O-), 4.06 (3H, s, -OCH_3_), 3.84 (2H, t, *J* = 6.5 Hz, ClCH_2_CH_2_CH_2_O-), 2.30 (2H, m, ClCH_2_CH_2_CH_2_O-); LC-MS: 413.0 (M+H)^+^; ^13^C-NMR (DMSO-*d_6_*) δ: 154.57 (C-7), 153.92 (C-6), 150.31 (C-10), 142.11 (C-9), 141.94, 134.28, 122.82, 120.06, 107.51 (C-5), 104.54 (C-4), 100.44 (C-8), 66.29 (ClCH_2_CH_2_CH_2_O-), 57.26 (-OCH_3_), 42.19 (ClCH_2_CH_2_CH_2_O-), 31.84 (ClCH_2_CH_2_CH_2_O-); Anal. Calcd. for C_17_H_15_Cl_3_N_4_O_2_: C 49.36, H 3.65, N 13.54; Found: C 49.28, H 3.66, N 13.56.

*7-(3-Chloropropoxy)-4-(3,4-dichlorophenyl)-6-methoxy-1,2,3-benzotriazine* (**8i**): Yield: 72.4%; mp: 120-122 ^o^C. IR (KBr): 3424, 1616, 1510, 1426, 1281, 848 cm^-1^; ^1^H-NMR (DMSO-*d_6_*) δ: 9.74 (1H, s, NH), 8.31 (1H, d, *J* = 2.31 Hz, H-2’), 7.93 (2H, m, H-8, H-6’), 7.71 (1H, m, H-5’), 7.67 (1H, s, H-5), 4.38 (2H, t, *J* = 6.0 Hz, ClCH_2_CH_2_CH_2_O-), 4.05 (3H, s, -OCH_3_), 3.84 (2H, t, *J* = 6.4 Hz, ClCH_2_CH_2_CH_2_O-), 2.30 (2H, m, ClCH_2_CH_2_CH_2_O-); LC-MS: 413.0 (M+H)^+^; ^13^C-NMR (DMSO-*d_6_*) δ: 154.11 (C-7), 153.43 (C-6), 149.95 (C-10), 141.66 (C-9), 139.23, 130.84, 130.51, 125.02, 123.14, 121.90, 107.10 (C-5), 104.11 (C-4), 100.08 (C-8), 65.86 (ClCH_2_CH_2_CH_2_O-), 56.85 (-OCH_3_), 41.87 (ClCH_2_CH_2_CH_2_O-), 31.46 (ClCH_2_CH_2_CH_2_O-); Anal. Calcd. for C_17_H_15_Cl_3_N_4_O_2_: C 49.36, H 3.65, N 13.54; Found: C 49.31, H 3.66, N 13.56.

*4-(4-Chloroanilino)-7-(3-chloropropoxy)-6-methoxy-1,2,3-benzotriazine* (**8j**): Yield: 67.9%; mp: 253 ^o^C. IR (KBr): 3425, 2925, 1616, 1561, 1495, 1424, 1245, 839 cm^-1^; ^1^H-NMR (DMSO-*d_6_*) δ: 9.66 (1H, s, NH), 7.91 (3H, m, H-8, H-2’, H-6’), 7.62 (1H, s, H-5), 7.50 (2H, d, *J* = 8.8 Hz, H-3’, H-5’), 4.37 (2H, t, *J* = 6.0 Hz, ClCH_2_CH_2_CH_2_O-), 4.04 (3H, s, -OCH_3_), 3.84 (2H, t, *J* = 6.4 Hz, ClCH_2_CH_2_CH_2_O-), 2.29 (2H, m, ClCH_2_CH_2_CH_2_O-); LC-MS: 379.0 (M+H)^+^; ^13^C-NMR (DMSO-*d_6_*) δ: 153.99 (C-7), 153.29 (C-6), 150.12 (C-10), 141.57 (C-9), 137.91 (C-1’), 128.59, 127.54, 123.99, 107.07 (C-5), 104.07 (C-4), 100.23 (C-8), 65.82 (ClCH_2_CH_2_CH_2_O-), 56.82 (-OCH_3_), 41.87 (ClCH_2_CH_2_CH_2_O-), 31.47 (ClCH_2_CH_2_CH_2_O-); Anal. Calcd. for C_17_H_16_Cl_2_N_4_O_2_: C 53.84, H 4.25, N 14.77; Found: C 53.79, H 4.24, N 14.71.

*7-(3-Chloropropoxy)-6-methoxy-4-(4-methylanilino)-1,2,3-benzotriazine* (**8k**): Yield: 70.1%; mp: 247 ^o^C. IR (KBr): 3420, 2924, 1615, 1513, 1425, 1282, 1242, 859 cm^-1^; ^1^H-NMR (DMSO-*d_6_*) δ: 9.55 (1H, s, NH), 7.92 (1H, s, H-8), 7.69 (2H, d, *J* = 8.4 Hz, H-2’, H-6’), 7.59 (1H, s, H-5), 7.25 (2H, d, *J* = 8.4 Hz, H-3’, H-5’), 4.37 (2H, t, *J* = 6.0 Hz, ClCH_2_CH_2_CH_2_O-), 4.03 (3H, s, -OCH_3_), 3.84 (2H, t, *J* = 6.3 Hz, ClCH_2_CH_2_CH_2_O-), 2.34 (3H, s, -CH_3_), 2.29 (2H, m, ClCH_2_CH_2_CH_2_O-); LC-MS: 359.1 (M+H)^+^;^ 13^C-NMR (DMSO-*d_6_*) δ: 154.23 (C-7), 153.51 (C-6), 150.62 (C-10), 141.59 (C-9), 136.43 (C-1’), 133.64, 129.48, 123.22, 107.28 (C-5), 104.42 (C-4), 100.86 (C-8), 66.14 (ClCH_2_CH_2_CH_2_O-), 57.22 (-OCH_3_), 42.24 (ClCH_2_CH_2_CH_2_O-), 31.84 (ClCH_2_CH_2_CH_2_O-), 20.99(Ph-CH_3_); Anal. Calcd. for C_18_H_19_ClN_4_O_2_: C 60.25, H 5.34, N 15.61; Found: C 60.28, H 5.33, N 15.56.

*7-(3-Chloropropoxy)-4-(4-fluoroanilino)-6-methoxy-1,2,3-benzotriazine* (**8l**): Yield: 71.2%; mp: 166 ^o^C. IR (KBr): 3437, 1620, 1510, 1428, 1284, 842 cm^-1^; ^1^H-NMR (DMSO-*d_6_*) δ: 9.63 (1H, s, NH), 7.91 (1H, s, H-8), 7.84 (2H, m, H-2’, H-6’), 7.61 (1H, s, H-5), 7.29 (2H, t, *J* = 8.7 Hz, H-3’, H-5’), 4.37 (2H, t, *J* = 6.0 Hz, ClCH_2_CH_2_CH_2_O-), 4.03 (3H, s, -OCH_3_), 3.84 (2H, t, *J* = 6.4 Hz, ClCH_2_CH_2_CH_2_O-), 2.29 (2H, m, ClCH_2_CH_2_CH_2_O-); LC-MS: 363.1 (M+H)^+^; ^13^C-NMR (DMSO-*d_6_*) δ: 160.67, 157.48, 154.28 (C-7), 153.56 (C-6), 150.64 (C-10), 141.79 (C-9), 135.46 (C-1’), 125.15, 125.04, 115.79, 115.50, 107.40 (C-5), 104.29 (C-4), 100.67 (C-8), 66.18 (ClCH_2_CH_2_CH_2_O-), 57.17 (-OCH_3_), 42.22 (ClCH_2_CH_2_CH_2_O-), 31.86 (ClCH_2_CH_2_CH_2_O-); Anal. Calcd. for C_17_H_16_ClFN_4_O_2_: C 56.28, H 4.45, N 15.44; Found: C 56.20, H 4.43, N 15.56.

*4-(3-Chloro-4-fluoroanilino)-7-(3-chloropropoxy)-6-methoxy-1,2,3-benzotriazine* (**8m**): Yield: 77.1%; mp: 138-139 ^o^C. IR (KBr): 3314, 2923, 1616, 1571, 1511, 1427, 1285, 849 cm^-1^; ^1^H-NMR (DMSO-*d_6_*) δ: 9.70 (1H, s, NH), 8.18 (1H, d, *J* = 4.4 Hz, H-2’), 7.90 (1H, s, H-8), 7.82 (1H, m, H-6’), 7.64 (1H, s, H-5), 7.51 (1H, t, *J* = 9.1 Hz, H-5’), 4.38 (2H, t, *J* = 5.9 Hz, ClCH_2_CH_2_CH_2_O-), 4.04 (3H, s, -OCH_3_), 3.84 (2H, t, *J* = 6.4 Hz, ClCH_2_CH_2_CH_2_O-), 2.29 (2H, m, ClCH_2_CH_2_CH_2_O-); LC-MS: 397.0 (M+H)^+^; ^13^C-NMR (DMSO-*d_6_*) δ: 155.47 (C-4’), 154.32 (C-7), 153.64 (C-6), 152.25 (C-4’), 150.32 (C-10), 141.81 (C-9), 136.57 (C-1’), 124.15 (C-2’), 122.93 (C-6’), 122.84 (C-6’), 119.47 (C-6’), 117.14 (C-5’), 116.85 (C-5’), 107.33 (C-5), 104.28 (C-4), 100.56 (C-8), 66.19 (ClCH_2_CH_2_CH_2_O-), 57.21 (-OCH_3_), 42.18 (ClCH_2_CH_2_CH_2_O-), 31.86 (ClCH_2_CH_2_CH_2_O-); Anal. Calcd. for C_17_H_15_Cl_2_FN_4_O_2_: C 51.40, H 3.81, N 14.10; Found: C 51.41, H 3.85, N 14.08.

*7-(3-Chloropropoxy)-6-methoxy-4-(3-trifluoromethoxyanilino)-1,2,3-benzotriazine* (**8n**): Yield: 65.3%; mp: 154-155 ^o^C. IR (KBr): 3427, 1615, 1572, 1511, 1448, 1256, 854 cm^-1^; ^1^H-NMR (DMSO-*d_6_*) δ: 9.75 (1H, s, NH), 8.05 (1H, s, H-2’), 7.93 (2H, s, H-8, H-6’), 7.65 (1H, s, H-5), 7.59 (1H, t, *J* = 8.2 Hz, H-5’), 7.15 (1H, d, *J* = 7.9 Hz, H-4’), 4.38 (2H, t, *J* = 5.9 Hz, ClCH_2_CH_2_CH_2_O-), 4.06 (3H, s, -OCH_3_), 3.84 (2H, t, *J* = 6.4 Hz, ClCH_2_CH_2_CH_2_O-), 2.29 (2H, m, ClCH_2_CH_2_CH_2_O-); LC-MS: 429.1 (M+H)^+^; ^13^C-NMR (DMSO-*d_6_*) δ: 154.34 (C-7), 153.65 (C-6), 150.40 (C-10), 148.82, 141.96 (C-9), 141.12 (C-1’), 130.54, 124.05, 122.30, 118.90, 115.91, 114.64, 107.32 (C-5), 104.41 (C-4),100.46 (C-8), 66.15 (ClCH_2_CH_2_CH_2_O-), 57.17 (-OCH_3_), 42.17 (ClCH_2_CH_2_CH_2_O-), 31.82 (ClCH_2_CH_2_CH_2_O-); Anal. Calcd. for C_1__8_H_1__6_ClF_3_N_4_O_3_: C 50.42, H 3.76, N 13.07; Found: C 50.37, H 3.78, N 13.06.

*7-(3-Chloropropoxy)-6-methoxy-4-(4-trifluoromethoxyanilino)-1,2,3-benzotriazine* (**8o**): Yield: 63.0%; mp: 150 ^o^C. IR (KBr): 3444, 1618, 1574, 1510, 1429, 1247, 852 cm^-1^; ^1^H-NMR (DMSO-*d_6_*) δ: 9.68 (1H, s, NH), 7.93 (2H, d, *J* = 9.0 Hz, H-2’, H-6’), 7.88 (1H, s, H-8), 7.59 (1H, s, H-5), 7.40 (2H, d, *J* = 8.7 Hz, H-3’, H-5’), 4.33 (2H, t, *J* = 5.9 Hz, ClCH_2_CH_2_CH_2_O-), 4.00 (3H, s, -OCH_3_), 3.79 (2H, t, *J* = 6.6 Hz, ClCH_2_CH_2_CH_2_O-), 2.25 (2H, m, ClCH_2_CH_2_CH_2_O-); LC-MS: 429.1 (M+H)^+^; ^13^C-NMR (DMSO-*d_6_*) δ: 154.41 (C-7), 153.70 (C-6), 150.54 (C-10), 144.48, 141.97 (C-9), 138.51 (C-1’), 125.64, 124.23, 122.34, 121.79, 107.46 (C-5), 104.43 (C-4), 100.68 (C-8), 66.23 (ClCH_2_CH_2_CH_2_O-), 57.22 (-OCH_3_), 42.21 (ClCH_2_CH_2_CH_2_O-), 31.85 (ClCH_2_CH_2_CH_2_O-); Anal. Calcd. for C_1__8_H_1__6_ClF_3_N_4_O_3_: C 50.42, H 3.76, N 13.07; Found: C 50.39, H 3.75, N 13.04.

*7-(3-Chloropropoxy)-4-(4-fluoro-3-trifluoromethylanilino)-6-methoxy-1,2,3-benzotriazine* (**8p**): Yield: 71.1%; mp: 150 ^o^C. IR (KBr): 3452, 2920, 1614, 1508, 1432, 1290, 1129 cm^-1^; ^1^H-NMR (DMSO-*d_6_*) δ: 9.82 (1H, s, NH), 8.18 (2H, m, H-6’, H-2’), 7.91 (1H, s, H-8), 7.65 (1H, s, H-5), 7.60 (1H, m, H-5’), 4.38 (2H, t, *J* = 6.0 Hz, ClCH_2_CH_2_CH_2_O-), 4.05 (3H, s, -OCH_3_), 3.84 (2H, t, *J* = 6.3 Hz, ClCH_2_CH_2_CH_2_O-), 2.30 (2H, m, ClCH_2_CH_2_CH_2_O-); LC-MS: 431.1 (M+H)^+^; ^13^C-NMR (DMSO-*d_6_*) δ: 156.36, 154.04 (C-7), 153.35 (C-6), 150.01 (C-10), 141.53 (C-9), 135.74 (C-1’), 128.37, 128.26, 124.46, 120.85, 120.26, 117.61, 117.33, 107.05 (C-5), 103.94 (C-4), 100.10 (C-8), 65.83 (ClCH_2_CH_2_CH_2_O-), 56.80 (-OCH_3_), 41.78 (ClCH_2_CH_2_CH_2_O-), 31.44 (ClCH_2_CH_2_CH_2_O-); Anal. Calcd. for C_18_H_15_ClF_4_N_4_O_2_: C 50.19, H 3.51, N 13.01; Found: C 50.17, H 3.53, N 13.00.

*7-(3-Chloropropoxy)-6-methoxy-4-(3-trifluoromethylanilino)-1,2,3-benzotriazine*(**8q**): Yield: 75.2%; mp: 180-181 ^o^C. IR (KBr): 3438, 2925, 1623, 1576, 1510, 1448, 1244, 799 cm^-1^; ^1^H-NMR (DMSO-*d_6_*) δ: 9.81 (1H, s, NH), 8.30 (1H, s, H-2’), 8.25 (1H, d, *J* = 8.4 Hz, H-6’), 7.94 (1H, s, H-8), 7.70 (1H, d, *J* = 7.8 Hz, H-5’), 7.66 (1H, s, H-5), 7.51 (1H, d, *J* = 8.1 Hz, H-4’), 4.38 (2H, t, *J* = 6.0 Hz, ClCH_2_CH_2_CH_2_O-), 4.06 (3H, s, -OCH_3_), 3.84 (2H, t, *J* = 6.4 Hz, ClCH_2_CH_2_CH_2_O-), 2.29 (2H, m, ClCH_2_CH_2_CH_2_O-); LC-MS: 413.1 (M+H)^+^; ^13^C-NMR (DMSO-*d_6_*) δ: 154.10 (C-7), 153.41 (C-6), 150.14 (C-10), 141.66 (C-9), 139.87 (C-1’), 129.88, 129.53, 129.32, 125.71, 119.93, 118.17, 107.09 (C-5), 104.11 (C-4), 100.14 (C-8), 65.85 (ClCH_2_CH_2_CH_2_O-), 56.83 (-OCH_3_), 41.84 (ClCH_2_CH_2_CH_2_O), 31.45 (ClCH_2_CH_2_CH_2_O-); Anal. Calcd. for C_18_H_16_ClF_3_N_4_O_2_: C 52.37, H 3.91, N 13.57; Found: C 52.32, H 3.94, N 13.56.

*4-Anilino-7-(3-chloropropoxy)-6-methoxy-1,2,3-benzotriazine* (**8r**): Yield: 70.1%; mp: 243 ^o^C. IR (KBr): 3435, 2922, 1614, 1566, 1502, 1447, 1421, 1285, 1241, 1108, 850, 744 cm^-1^; ^1^H-NMR (DMSO-*d_6_*) δ: 9.59 (1H, s, NH), 7.94 (1H, s, H-8), 7.84 (2H, d, *J* = 7.8 Hz, ArH-2’H, 6’H), 7.61 (1H, s, H-5), 7.45 (2H, t, *J* = 7.8 Hz, ArH-3’H, 5’H), 7.18 (1H, t, *J* = 7.5 Hz, ArH-4’H), 4.37 (2H, t, *J* = 6.0 Hz, ClCH_2_CH_2_CH_2_O-), 4.04 (3H, s, -OCH_3_), 3.84 (2H, t, *J* = 6.3 Hz, ClCH_2_CH_2_CH_2_O-), 2.29 (2H, m, ClCH_2_CH_2_CH_2_O-); LC-MS: 345.2 (M+H)^+^;^ 13^C-NMR (DMSO-*d_6_*) δ: 153.88 (C-7), 153.17 (C-6), 150.28 (C-10), 141.50 (C-9), 138.83 (C-1’), 128.68 (C-3’, C-5’), 124.03 (C-4’), 122.88 (C-2’, C-6’), 107.01 (C-5), 104.02 (C-4), 100.31 (C-8), 65.78 (ClCH_2_CH_2_CH_2_O-), 56.81 (-OCH_3_), 41.89 (ClCH_2_CH_2_CH_2_O-), 31.48 (ClCH_2_CH_2_CH_2_O-); Anal. Calcd. for C_17_H_17_ClN_4_O_2_: C 59.22, H 4.97, N 16.25; Found: C 59.28, H 4.96, N 16.17.

### Antiproliferative activity assay

*Cell lines*: The cell lines used were obtained from ATCC (Rockville, MD, USA). T47D human breast cancer cells, DU145 and PC-3 human prostate cancer cells were cultured in RPMI1640 medium (Gibco; New York, NY, USA) supplemented with 100 units/mL penicillin, 100 µg/mL streptomycin, 1 mM L-glutamine, 5 µg/mL insulin and 10% (v/v) heat-inactivated fetal bovine serum (FBS). MVECs, LL/2 murine lewis lung carcinoma cells and B16F0 melanoma cells were cultured in DMEM with 4 mM L-glutamine adjusted to contain 4.50 g/L glucose and 10% (v/v) heat-inactivated FBS.

*Cell growth inhibition assay*: The antiproliferative activities of these compounds were determined by the MTT assay. Cells (2×10^3^ cells/well in 96-well plates) were incubated for 24 h, then various concentrations of each compound were added to each well and the cells were cultured for another 4 days at 37 ^o^C. MTT solution (50 μL of 2 mg/mL) was added per well and the culture continued for an additional 4 h. The medium was removed by aspiration and the cells were dissolved in 200 μL DMSO. The absorbance at 570 nm was measured in the 96-well plate reader. Growth inhibition is reported as compared to untreated cells (%) and GI_50_ concentration was calculated [18].

## References

[1] Ferrara N. (2004). Vascular endothelial growth factor as a target for anticancer therapy. Oncologist.

[2] Poon R. T., Fan S. T., Wong J. (2001). Clinical implications of circulating angiogenic factors in cancer patients. J. Clin. Oncol..

[3] Toi M., Matsumoto T., Bando H. (2001). Vascular endothelial growth factor: its prognostic, predictive, and therapeutic implications. Lancet Oncol..

[4] Wood J. M., Bold G., Buchdunger E., Cozens R., Ferrari S., Frei J., Hofmann F., Mestan J., Mett H., O’Reilly T., Persohn E., Rosel J., Schnell C., Stover D., Theuer A., Towbin H., Wenger F., Woods-Cook K., Menrad A., Siemeister G., Schirner M., Thierauch K.-H., Schneider M. R., Drevs J., Martiny-Baron G., Totzke F., Marme D. (2000). PTK787/ZK 222584, a novel and potent inhibitor of vascular endothelial growth factor receptor tyrosine kinases, impairs vascular endothelial growth factorinduced responses and tumor growth after oral administration. Cancer Res..

[5] Bold G., Altmann K. H., Frei J., Lang M., Manley P. W., Traxler P., Wietfeld B., Bruggen J., Buchdunger E., Cozens R., Ferrari S., Furet P., Hofmann F., Martiny-Baron G., Mestan J., Rosel J., Sills M., Stover D., Acemoglu F., Boss E., Emmenegger R., Lasser L., Masso E., Roth R., Schlachter C., Vetterli W. (2000). New anilinophthalazines as potent and orally well absorbed inhibitors of the VEGF receptor tyrosine kinases useful as antagonists of tumor-driven angiogenesis. J. Med. Chem..

[6] Heymach J. V. (2005). ZD6474--clinical experience to date. Brit. J. Cancer.

[7] Sahtornsumetee S., Rich J. N. (2006). Vandetanib (ZD6474), a novel multitargeted kinase inhibitor, in cancer therapy. Drugs Today.

[8] Joensuu H., De Braud F., Coco P., De Pas T., Putzu C., Spreafico C., Bono P., Bosselli S., Jalava T., Laurent D., Casali P. G. (2008). Phase II, open-label study of PTK787/ZK222584 for the treatment of metastatic gastrointestinal stromal tumors resistant to imatinib mesylate. Ann. Oncol..

[9] Thomas A. L., Trarbach T., Bartel C., Laurent D., Henry A., Poethig M., Wang J., Masson E., Steward W., Vanhoefer U., Wiedenmann B. (2007). A phase IB, open-label dose-escalating study of the oral angiogenesis inhibitor PTK787/ZK 222584 (PTK/ZK), in combination with FOLFOX4 chemotherapy in patients with advanced colorectal cancer. Ann. Oncol..

[10] Kovacs M. J., Reece D. E., Marcellus D., Meyer R. M., Mathews S., Dong R. P., Eisenhauer E. (2006). A phase II study of ZD6474 (Zactima, a selective inhibitor of VEGFR and EGFR tyrosine kinase in patients with relapsed multiple myeloma--NCIC CTG IND.145. Invest. New Drugs.

[11] Lee D. (2005). Phase II data with ZD6474, a small-molecule kinase inhibitor of epidermal growth factor receptor and vascular endothelial growth factor receptor, in previously treated advanced non-small-cell lung cancer. Clin. Llung Cancer.

[12] Miller K. D., Trigo J. M., Wheeler C., Barge A., Rowbottom J., Sledge G., Baselga J. (2005). A multicenter phase II trial of ZD6474, a vascular endothelial growth factor receptor-2 and epidermal growth factor receptor tyrosine kinase inhibitor, in patients with previously treated metastatic breast cancer. Clin. Cancer Res..

[13] Gueto C., Ruiz J. L., Torres J. E., Méndez J., Vivas-Reyes R. (2008). Three-dimensional quantitative structure-activity relationship studies on novel series of benzotriazine based compounds acting as Src inhibitors using CoMFA and CoMSIA. Bioorg. Med. Chem..

[14] Noronha G., Barrett K., Cao J., Dneprovskaia E., Fine R., Gong X., Gritzen C., Hood J., Kang X., Klebansky B., Li G., Liao W., Lohse D., Mak C. C., McPherson A., Palanki M. S., Pathak V. P., Renick J., Soll R., Splittgerber U., Wrasidlo W., Zeng B., Zhao N., Zhou Y. (2006). Discovery and preliminary structure-activity relationship studies of novel benzotriazine based compounds as Src inhibitors. Bioorg. Med. Chem. Lett..

[15] Wissner A., Floyd M. B., Johnson B. D., Fraser H., Ingalls C., Nittoli T., Dushin R. G., Discafani C., Nilakantan R., Marini J., Ravi M., Cheung K., Tan X., Musto S., Annable T., Siegel M. M., Loganzo F. (2005). 2-(Quinazolin-4-ylamino)-[1,4]benzoquinones as covalent-binding, irreversible inhibitors of the kinase domain of vascular endothelial growth factor receptor-2. J. Med. Chem..

[16] Siddiqui M. S. S., Stevens M. F. G. (1974). Triazines and related products. Part XI. Dimroth rearrangements of 3-substituted 3,4-dihydro-4-imino-1,2,3-benzotriazines in acetic acid. J. Chem. Soc., Perkin Trans. 1.

[17] Hess-Stumpp H., Haberey M., Thierauch K. H. (2005). PTK 787/ZK 222584, a tyrosine kinase inhibitor of all known VEGF receptors, represses tumor growth with high efficacy. Chembiochem.

[18] Lu M., Xia L., Luo D., Waxman S., Jing Y. (2004). Dual effects of glutathione-S-transferase pi on As2O3 action in prostate cancer cells: enhancement of growth inhibition and inhibition of apoptosis. Oncogene.

